# Supplementary material for: The Evolution and Characterization of the RNA Interference Pathways in Lophotrochozoa
Source: Genome Biol Evol. 2024 May 7;16(5):evae098. doi: 10.1093/gbe/evae098 (PMC11114477; doi:10.1093/gbe/evae098)
Supplement: evae098_Supplementary_Data [file evae098_supplementary_data.zip › Supplementary_materials.zip!/selected_miRNAs/NoGe_selected_miRNAs/mirdeep_pdf_files/NMRB01000004.1_Notospermus_geniculatus_strain_Ushimado_scaffold4,_whole_genome_shotgun_sequence_164.pdf]

Provisional ID : NMRB01000004.1_Notospermus_geniculatus_strain_Ushimado_scaffold4,_whole_genome_shotgun_sequence_164

Score total	:	106.8
Score for star read(s)	:	3.9
Score for read counts	:	102.1
Score for mfe	:	1.4
Score for randfold	:	0
Score for cons. seed	:	-0.6
Total read count	:	212
Mature read count	:	99
Loop read count	:	0
Star read count	:	113

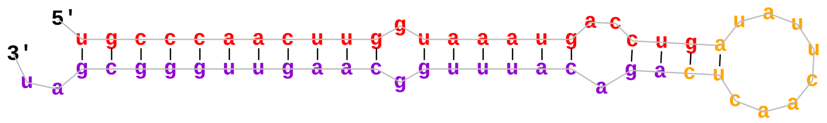

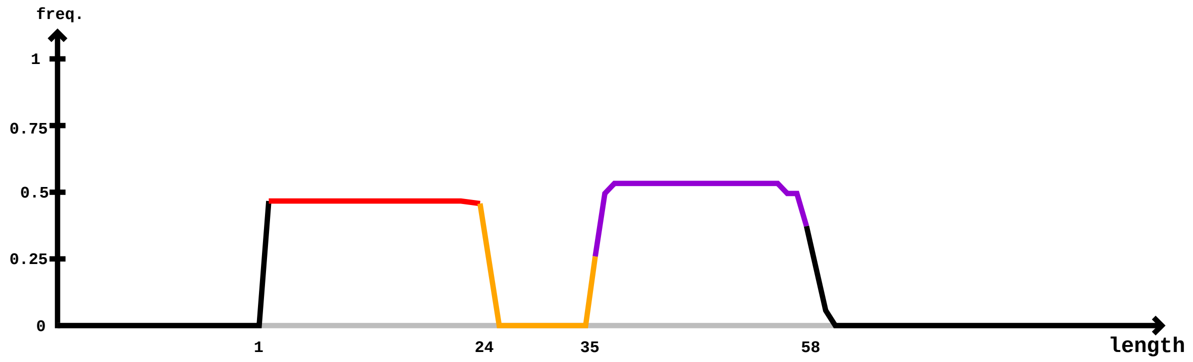


```
                   Mature                           Star
5'- caauuugauccuuuagugcuugcccaacuugguaaaugaccugauauucaacucagacauuuggcaaguugggcgauuucuuuggguuugaacuugcugaaacacacaaaaga   -3'    obs
    caauuugauccuuuagugcuugcccaacuugguaaaugaccugauauucaacucagacauuuggcaaguugggcgauuucuuuggguuugaacuugcugaaacacacaaaaga          exp
    ((((((((.(((..((...((((((((((((.((((((..((((........)))).)))))).))))))))))))...))..))).))))).))).................    reads     mm      sample
    ....................ugcccaacuugguaaCugacc........................................................................    1         1       Mm3
    ....................ugcccaacuugguaaCugaccuga.....................................................................    1         1       Mm3
    ....................ugcccaacuugguaaaugaccuga.....................................................................    1         0       Mm3
    ......................................................agacauuuggcaaguugggc.......................................    1         0       Mm3
    ......................................................agacauuuggcaaguugggcga.....................................    1         0       Mm3
    ......................................................agacauuuggcaaguugggcgau....................................    2         0       Mm3

    ....................ugcccaacuugguaaCugaccug......................................................................    2         1       Fm3
    ....................ugcccaacuugguaaCugaccuga.....................................................................    7         1       Fm3
    ......................................................agacauuuggcaaguugggcgau....................................    6         0       Fm3
    ......................................................agacauuuggcaaguugggcgaAu...................................    2         1       Fm3
    ......................................................agacauuuggcaaguugggcgauA...................................    2         1       Fm3
    .......................................................gacauuuggcaaguugggc.......................................    6         0       Fm3
    .......................................................gacauuuggcaaguuggUcga.....................................    1         1       Fm3
    .......................................................gacauuuggcaaguugggcga.....................................    12        0       Fm3
    .......................................................gacauuuggcaaguugggcgau....................................    15        0       Fm3
    .......................................................gacauuuggcaaguugggcgauu...................................    3         0       Fm3
    ........................................................acauuuggcaaguugggcga.....................................    5         0       Fm3

    ....................ugcccaacuugguaaaugaccug......................................................................    8         0       Mm1
    ......................................................agacauuuggcaaguugAgcgau....................................    1         1       Mm1
    ......................................................agacauuuggcaaguugCgcgau....................................    1         1       Mm1
    ......................................................agacauuuggcaaguugggcgau....................................    8         0       Mm1
    ......................................................agacauuuggcaGguugggcgauu...................................    4         1       Mm1
    .......................................................gacauuuggcaaguugggcgaAu...................................    9         1       Mm1

    ....................ugcccaacuugguaaaugaccu.......................................................................    1         0       Mm2
    ....................ugcccaacuugguaaaugaccug......................................................................    43        0       Mm2
    ....................ugcccaacuugguaaCugaccug......................................................................    5         1       Mm2
    ....................ugcccaacuugguaaaugaccuga.....................................................................    11        0       Mm2
    ....................ugcccaacuugguaaaugaccugU.....................................................................    8         1       Mm2
    ....................ugcccaacuugguaaCugaccuga.....................................................................    5         1       Mm2
    ......................................................agacauuuggcaaguugggcgU.....................................    1         1       Mm2
```

```
                    Mature                           Star
caauuugauccuuuagugcuugcccaacuugguaaaugaccugauauucaacucagacauuuggcaaguugggcgauuucuuggguuugaacuugcugaaacacacaaaaga
......................................................agacauuuggcaaguugggcga....................................    6    0    Mm2
......................................................agacauuuggcaaguugggcgau...................................    2    0    Mm2
......................................................agacauuuggcaaguugggcgauu..................................    1    0    Mm2
.......................................................gacauuuggcaaguugggc......................................    1    0    Mm2
.......................................................gacauuuggcaaguugggcgau...................................    3    0    Mm2

....................ugcccaacuugguaaaugaccugU....................................................................    6    1    Fm2
......................................................agacauuuggcaaguugggcgauuu.................................   12    0    Fm2
........................................................acauuuggcaaguugggcgauA..................................    3    1    Fm2

......................................................agacauuuggcaaguugggcgau...................................    5    0    Fm1
```
